# Consensus analysis via weighted gene co-expression network analysis (WGCNA) reveals genes participating in early phase of acute respiratory distress syndrome (ARDS) induced by sepsis

**DOI:** 10.1080/21655979.2021.1909961

**Published:** 2021-04-05

**Authors:** Qing Fang, Qilai Wang, Zhiming Zhou, An Xie

**Affiliations:** Department of Pulmonary Medicine, HwaMei Hospital, University of Chinese Academy of Sciences,Ningbo Institute of Life and Health Industry,University of Chinese Academy of Scienc, Ningbo, Zhejiang, China

**Keywords:** WGCNA, ards, sepsis, gsva, consensus analysis

## Abstract

The understanding of mechanism during conversion from sepsis to sepsis-related ARDS remains limited. In this study, we collected gene expression matrix from the Gene Expression Omnibus (GEO) database and constructed networks using weighted gene co-expression network analysis (WGCNA) to identify the consensus and opposite modules between sepsis and sepsis-induced ARDS and obtained 27 consensus modules associated with sepsis and sepsis-related ARDS, including one model (160 genes) with opposite correlation and 1 sepsis-ARDS specific model with 34 genes. Differentially expressed genes analysis, functional enrichment and protein-protein interactions analyses of candidate genes were performed; 15 of these genes showed different expressions in sepsis-induced ARDS patients, compared with sepsis patients; genes were enriched in processes associated with ribosome, tissue mechanics and extracellular matrix. Feature selection analysis revealed that three genes, *TLCD4, PRSS30P*, and *ZNF493*, showed moderate performance in identifying sepsis-induced ARDS from sepsis. Ribosome-related genes indicate crucial roles in the development of sepsis-induced ARDS.

## Graphical abstract

**Figure:**
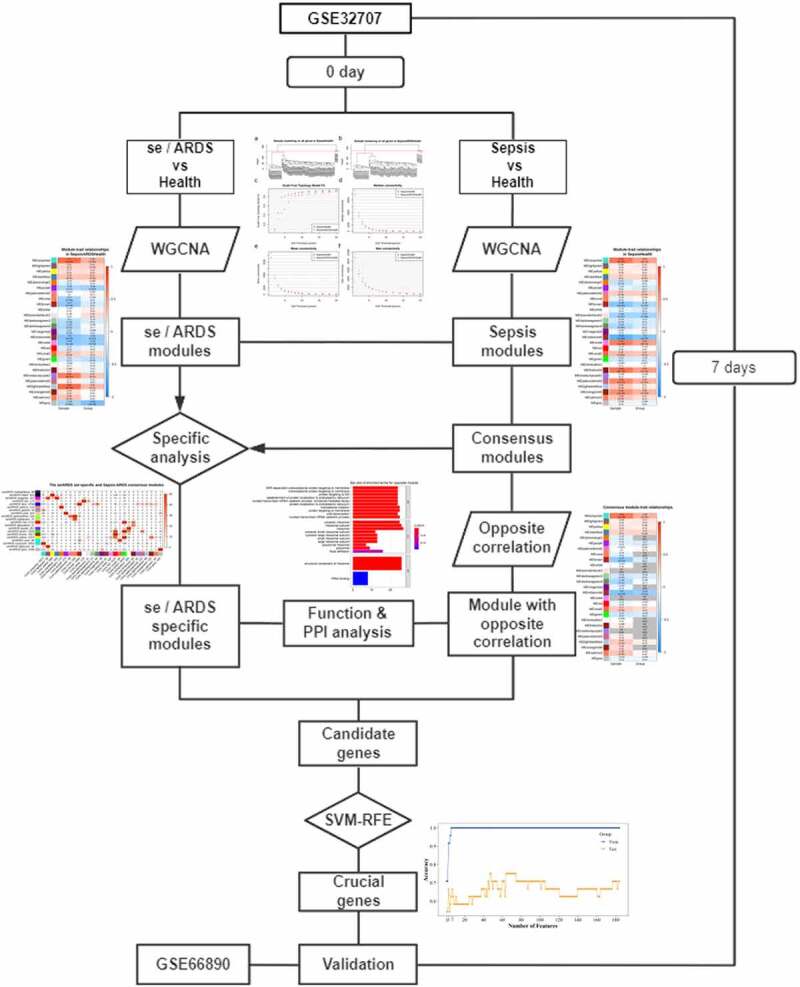

## 1. Introduction

Sepsis is a severe infectious condition that can result in immune system responses and organ dysfunction [1]. Acute respiratory distress syndrome (ARDS) is a major lung injury in intensive care unit (ICU) patients with a high mortality rate [2]. This adverse outcome is often due to an inflammatory response that influences fluid leakage and leukocyte recruitment into air spaces, thus causing hypoxemia [3]. Among the factors that contribute to the development of ARDS, sepsis is the most common cause. Sepsis-induced ARDS has a higher overall disease severity, poorer recovery from a lung injury, lower successful extubation rates, and a higher mortality rate compared with non-sepsis-induced ARDS [4].

There are numerous publications related to sepsis and sepsis-related ARDS. Increased expression of several genes in neutrophil-related pathways may be involved in the early pathogenesis of sepsis-induced ARDS [5]. Experimental data suggests that intravenous vitamin C may alleviate the inflammation and vascular injury related to sepsis and ARDS [2]. Multipotent mesenchymal stem (stromal) cells might decrease lung injury and enhance lung repair in sepsis and ARDS [6]. The correlation analysis showed that omega-3 fatty acids could reduce the death rate of sepsis and sepsis-induced ARDS [7]. Plasma angiopoietin-2, a vascular permeability marker, may be involved in ARDS development and can be used to treat sepsis-related ARDS [8]. *MYC* and *STAT3* may be the critical regulatory genes for the underlying dysfunction of sepsis-induced ARDS [9]. Studies on molecular biomarkers used for identifying ARDS from sepsis and genes related to incipient sepsis patients developing ARDS, however, are limited.

Weighted gene co-expression network analysis (WGCNA) is an effective method to identify the significant modules and hub genes associated with phenotypes [10]. It is a data reduction method, which can classify genes into a model based on pairwise correlations due to their similar expression profiles [11,12]. WGCNA is a comprehensive collection of R functions for conducting diverse aspects of weighted correlation network analysis. It has been used extensively in sepsis. It was used to explore the probable regulatory relationships of N6-methyladenosine in sepsis [13]. WGCNA was performed to identify the putative biomarkers in sepsis co-expression gene modules [14–16].

In this study, we constructed networks with WGCNA, performed consensus analysis between sepsis patients and sepsis-related ARDS patients, and obtained 27 consensus modules related to sepsis and sepsis-induced ARDS. Differentially expressed genes were analyzed in the identified modules. Functional enrichment analysis and protein–protein interactions were conducted. The candidate genes were evaluated with support vector machine recursive feature elimination method (SVM-RFE). We observed the critical role of ribosomal genes in the development of sepsis-related ARDS; three genes indicated potential, though not great, in identifying sepsis-induced ARDS from sepsis.

## 2. Materials and methods

### 2.1 Data collection and quality control

To explore the genes related to sepsis-induced ARDS, we collected and integrated the expression matrix GSE32707, which contained 58 sepsis patients (30 for 0 days and 28 for 7 days after admission), 31 sepsis-induced ARDS samples (18 for 0 days and 13 for 7 days), 21 SIRS (systemic inflammatory response syndrome) patients (0 days) and 34 control whole blood samples from the GEO database (https://www.ncbi.nlm.nih.gov/geo/). We annotated the expression matrix with its corresponding annotation file – GPL10558, Illumina HumanHT-12 V4.0 expression beadchip. Considering the impact of inflammation facts, we chose sepsis and sepsis-induced ARDS patients with 0 days after admission for this study. To validate our findings, we collected samples from another independent analysis – GSE66890, including 57 samples (29 sepsis-related ARDS patients and 28 sepsis patients within 24 hours after admission) and annotated them with the expression matrix with their related annotation file – GPL6244, Affymetrix Human Gene 1.0 ST Array (transcript (gene) version). We defined the average as the expression value of genes with multiple probes; normalized the matrix using the *limma* package with the ‘*quantile*’ method [17]; filtered for genes with missing values; and constructed the hierarchical clustering tree to identify and trim the outliers.

### 2.2 WGCNA network construction

We utilized WGCNA to explore genes associated with sepsis-induced ARDS. In detail, the ‘Group’ (sepsis, sepsis-induced ARDS, and healthy controls) information for samples, without outliers, was collected as clinical traits for this analysis [10,18]. The soft-thresholding power network topology analysis was performed; the suitable power value was used to construct the network; we calculated and transformed adjacencies into consensus Topological Overlap Matrix (TOM); considering the diverse statistical properties between different data sets, we scaled and transformed the sepsis TOM to make it equivalent to that of sepsis-induced ARDS and obtained consensus modules between two diseases; we depicted a quantile-quantile plot to visualize the improvement before and after scaling; the consensus TOM was calculated with component-wise (‘parallel’) minimum of the TOMs for each set. To obtain large modules, the ‘minModuleSize’ parameter, indicating the minimum module size of the modules, was set as 20. Genes with similar expression patterns were separated into different modules with the ‘cutreeDynamic’ function; to evaluate and group the co-expression similarities of all modules, the eigengenes (MEs) were calculated, clustered, and mapped to the related consensus modules; then, modules with a correlation of 0.75 were merged with ‘mergeCloseModules’ function using default parameters. To identify significant modules related to clinical traits, the association of clinical information, Gene Significance (GS), and Module membership (MM) was evaluated. The correlation between clinical traits and expression of samples was calculated using the ‘cor’ function; the *P*-value was calculated using the ‘corPvalueStudent’ function. To evaluate specific modules related to sepsis-induced ARDS, we performed network construction and module detection of sepsis-related ARDS and linked the detected modules of ARDS to the consensus modules.

### 2.3 Differentially expressed genes (DEGs) analysis

To explore expression patterns of candidate genes, we collected and established the expression matrix of candidate genes gained from WGCNA. The differentially expressed genes analysis (DEGA) was performed with the built matrix, normalized by *limma* package, and filtered with a hierarchical clustering tree. We constructed the contradicted matrix with clinical traits (sepsis and sepsis-induced ARDS) and analyzed DEGs with *limma* package; we defined the DEGs with the parameters (fold change ≠ 0 and *p*-value <0.05).

### 2.4 Functional analysis

The clusterProfiler, a comprehensive functional R package to understand the biological meaning, was utilized to perform functional enrichment analysis and visualization of candidate genes obtained from WGCNA [19]. The official gene symbols of the candidate genes were transformed into ‘ENTREZID’ and ‘UNIPROT’ according to annotation profile for human, for Gene Ontology (GO) and Kyoto Encyclopedia of Genes and Genomes (KEGG) enrichment analysis; all genes used for WGCNA analysis were utilized as background; the cutoff point for p-value and q-value was set to 10^(−4)^; ‘BH’ was used to adjust *p*-value; the minimal size of the annotated genes was 10. Significantly enriched terms of GO and KEGG were collected and visualized with bar plots.

### 2.5 Protein–protein interactions analysis

Candidate genes obtained from WGCNA were submitted to an online tool STRING (https://string-db.org/) to explore the interactions among proteins. Then the Cytoscape software was used to depict and integrate the interaction network; ‘MCODE’, a plugin of Cytoscape was applied to predict gene clusters; critical genes were collected using the ‘Degree’ method based on the calculation and ranking of the interactions among proteins.

### 2.6 Evaluation of candidate genes

To evaluate the potential of the candidate genes identified from WGCNA in distinguishing sepsis-induced ARDS patients and sepsis patients, we utilized support vector machine recursive feature elimination method (SVM-RFE), one of the most effective methods in filtering critical characters, to select crucial genes. Samples were divided into ‘train’ and ‘test’ sets (1:1) randomly; support vector machine (SVM) analysis was implemented with ‘e1071ʹ package. Their potential was validated with expression of patients on 7 days after admission in GSE32707 and another expression matrix, GSE66890.

### 2.7 Statistical methods

Consistency between arrays was performed with *limma* package; outliers were detected and removed with hierarchical cluster analysis in R software (https://www.r-project.org/). The correlation of models and clinical traits was calculated with ‘Pearson’ using R software; fold change, t-statistics and statistical significance of genes were calculated with *limma* package.

## 3. Results

### 3.1 Consensus modules and genes associated with sepsis and sepsis-related ARDS

**Figure 1.**
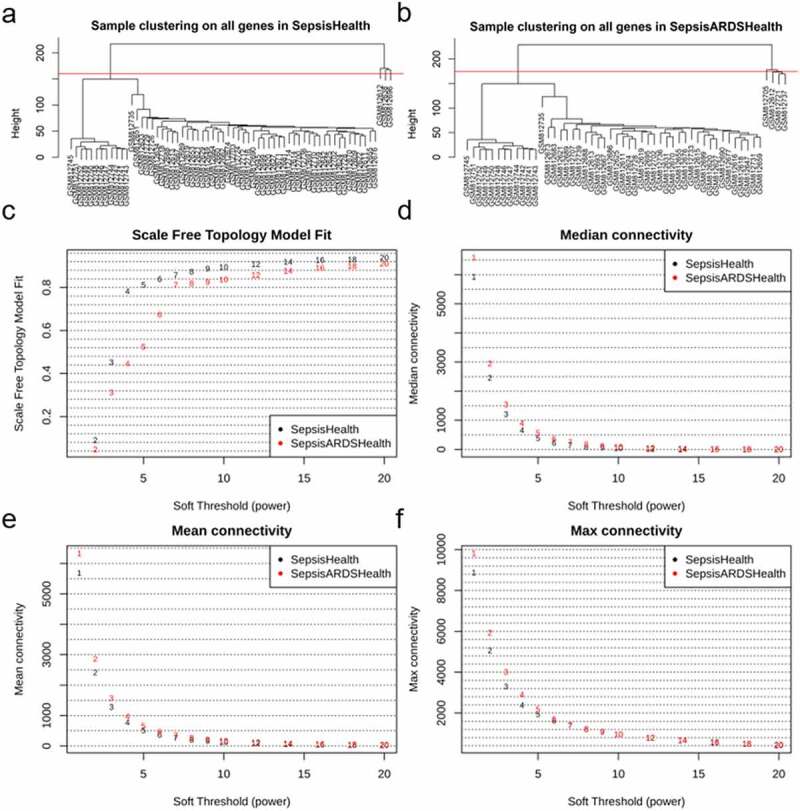
Identifying outliers and defining soft-thresholding power. (a) and (b) show the hierarchical clustering tree of two samples matrix; the red line indicates the cut position; samples above the red line will be discarded. (c-f) show the decision of power value; c, the scale-free fit index (y-axis) under different power (x-axis); (d) to (f), the median, mean, and max connectivity (y-axis) drops with the increase of the soft-thresholding power (x-axis). the plots indicate that the power of 7 is the suitable value because the connectivity measures decline steeply with the increase of power

**Figure 2.**
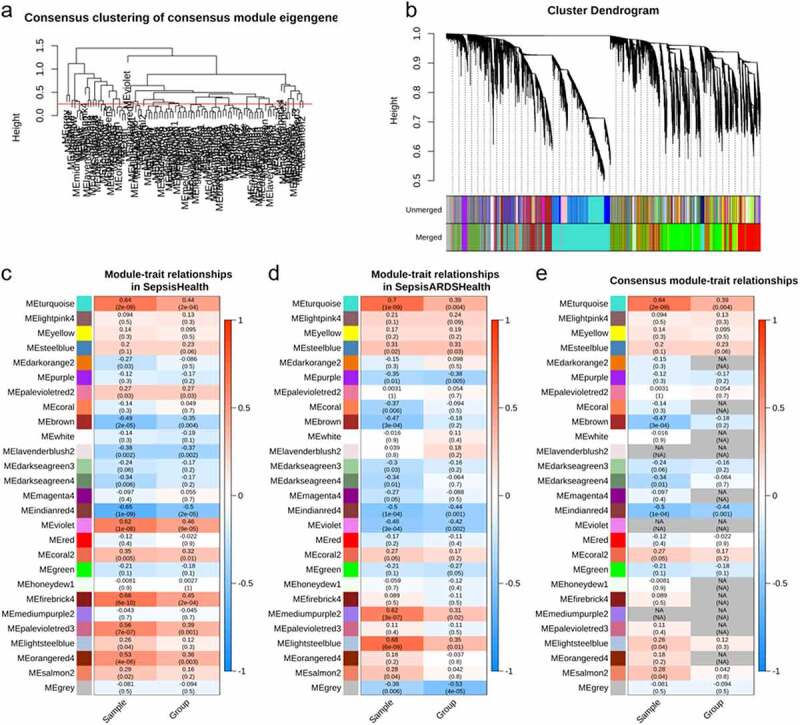
**Network construction of consensus modules**. (a) shows the clustering tree of module eigengenes; red line (0.25) represents that the correlation is 0.75 and modules under the line will be merged. (b), the clustering tree of genes (top), primary modules (middle), and merged modules (bottom); (c-e) relationships of consensus module eigengenes and clinical traits in sepsis, sepsis-induced ARDS, and across sepsis and sepsis-induced ARDS (se/ARDS) data. the numbers in the box indicate the correlation (top) and its *P*-value (bottom) of module eigengenes (rows) and clinical traits (columns). missing (NA) entries represent that the failure of formation of consensus because the correlations in sepsis and se/ARDS are opposite

The potential outliers will influence downstream analysis and even mislead us with confusing results. To remove impacts of outgroup sample, we performed hierarchical cluster analysis. Based on the clustering tree, we observed three outliers – GSM812612 (control), GSM812638 (sepsis patients, 0 days), and GSM812696 (sepsis patients, 0 days) (Figure 1a, 1b); we marked and discarded outliers – samples above the red line (Figure 1a, 1b) – and plotted the tree, once again, to ensure that there were no outliers. A total of 79 samples (28 sepsis patients, 18 sepsis-induced ARDS patients, and 33 healthy controls) with 20,919 genes were used in this study. We chose 7 as the suitable soft-thresholding power for each set in this analysis based on two criteria: the lowest power at which the scale-free topology fit index reaches 0.80; connectivity measurements decrease considerably (Figure 1c, 1d, 1e, 1f). We merged and obtained a total of 27 consensus gene co-expression modules (Supplementary File), with the number of genes ranging from 23 to 5973 (Figure 2b, 1b); the gray module containing 430 genes could not be assigned to any modules.

### 3.2 Relating consensus modules to sepsis and sepsis-related ARDS

The tables of module-trait relationships indicated the relation between the clinical traits (sepsis and control in Figure 2c, sepsis-related ARDS and control in Figure 2d) and the consensus modules in each data set. Two relation tables exhibit some degree of similarity. To explain further, turquoise, steelblue, and indianred4 module showed significant relations to clinical traits in each matrix, although the actual correlations and p-values of two data sets differed slightly. Some modules, such as the violet module, presented the opposite correlation with clinical traits. The similarity and difference were integrated and depicted in the comparable tables (Figure 2e). We kept the lower absolute value in two sets with the same sign of correlations, and zero (NA) for those with the opposite trend. From the comparable table, we found that the violet module containing 160 genes was significantly related to sepsis and sepsis-induced ARDS with the opposite trend. Fifteen of them (*KMO, RPS27A, SPNS2, TUBA1B, BASP1, FBRSL1, AATK, PLK5, LRRC75A, TLCD4, IP6K3, DPY19L2P2, LRRN2, BTLA*, and *ALOX15*) showed different expression in sepsis with ARDS compared to sepsis alone.

### 3.3 Functional enrichment of genes in the opposite module

**Figure 3.**
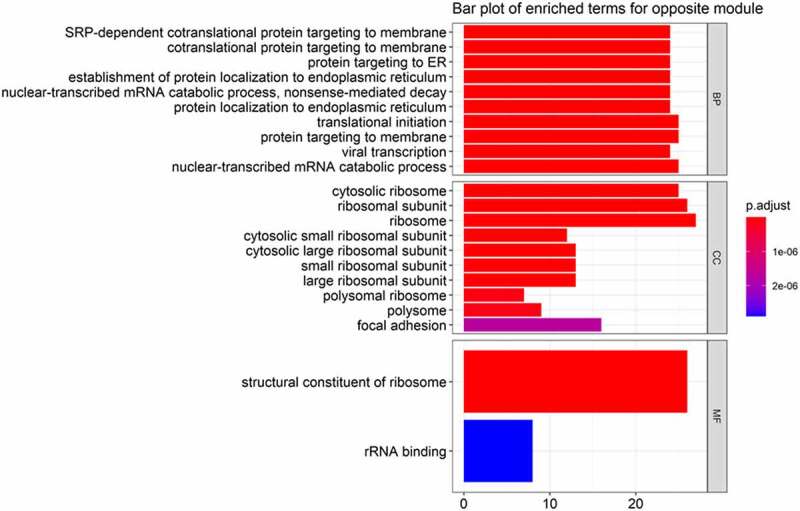
Bar plots for functional enrichment of genes in the opposite module. Top 10 GO terms in biological process (BP), cellular component (CC), and molecular function (MF) were depicted via bar plot

We obtained 160 genes significantly (*P* < 0.05) associated with sepsis and sepsis-induced ARDS with opposite signs (0.46 for sepsis and −0.42 for ARDS caused by sepsis). The candidate genes significantly (*P* < 10^−4^) enriched in 35 GO terms: 22 in biological process (BP), 11 in cellular component (CC), and 2 in molecular function (MF); the terms in each category were depicted with bar plots (Figure 3). One KEGG term – Ribosome (hsa03010) containing 24 genes (*RPL4, RPL3, RPL32, RPL10, RPS8, RPL12, RPL11, RPL13A, RPL23A, RPL6, RPS15, RPS4X, RPS14, RPL7A, RPS25, RPS28, RPS15A, RPS16, RPS27, RPL18A, RPS18, RPL27A, RPS2*, and *RPS27A*) – was significantly enriched (*P* < 10^−4^).

### 3.4 Protein–protein interactions of genes in the opposite module

**Figure 4.**
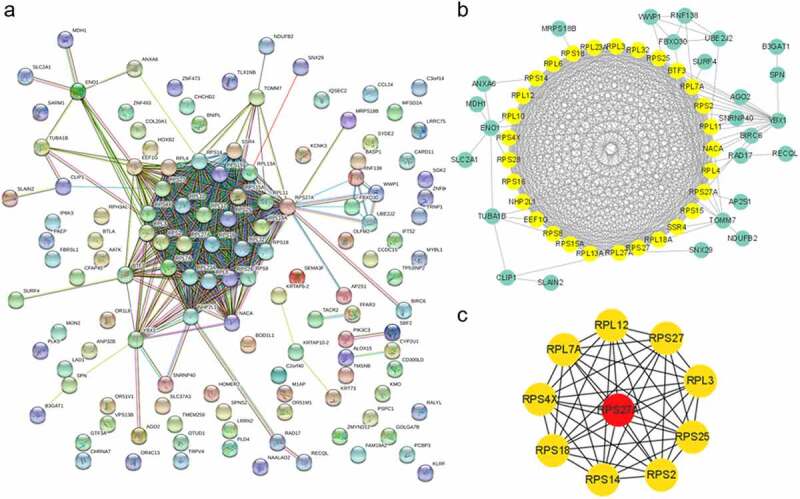
Protein-protein interactions of genes in the opposite module. (a) The image of PPI of genes in the opposite module. (b) The predicted genes cluster based on MCODE of cytoscape. (c) Top 10 hub genes predicted with ‘degree’ method of cytoscape

The interactions among protein genes provide a perceptive of genes working together. The interactions obtained from the STRING database indicated that the potential genes cluster were in the opposite module (Figure 4a). The genes in the predicted genes cluster by Cytoscape software were visualized with yellow color (Figure 4b). Ten hub genes were predicted and depicted by Cytoscape, as showed in the chart (Figure 4c).

### 3.5 Detecting specific modules associated with sepsis-induced ARDS

**Figure 5.**
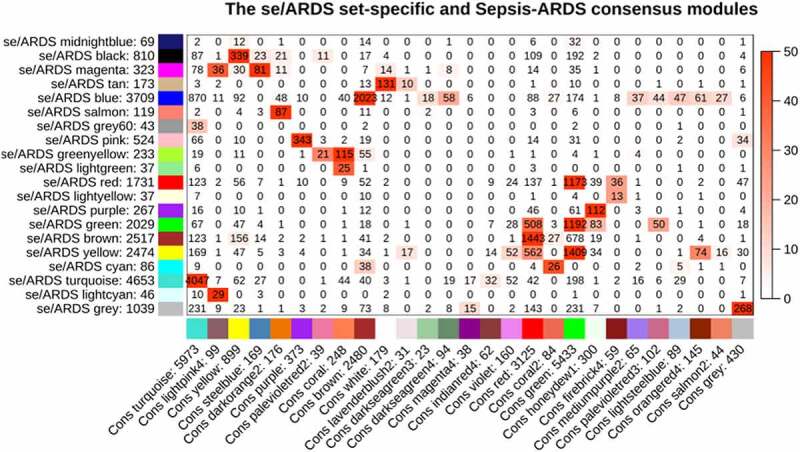
Correspondence of sepsis-induced (se/ARDS) set-specific and the se/ARDS-sepsis consensus modules. each row of the table refers to one se/ARDS set-specific module, each column to one consensus module. numbers indicate gene counts in the intersection of consensus modules. the significance of overlap is color-coded; the stronger the red color, the more significant the overlap is. the table represents that most se/ARDS set-specific modules are observed in consensus modules

Genes in specific modules associated with sepsis-induced ARDS usually play a crucial role in the development of ARDS. From the color-coded table (Figure 5), we observed that most modules in sepsis-induced ARDS had counterparts in the consensus module. This indicated the similarity and consensus of the genes in response to sepsis and sepsis-related ARDS. Interestingly, we found that 34 genes in the sepsis-induced sepsis (se/ARDS) set-specific module located in the grey consensus module.

### 3.6 PPI and functional analysis of the specific module related to ARDS with sepsis

**Figure 6.**
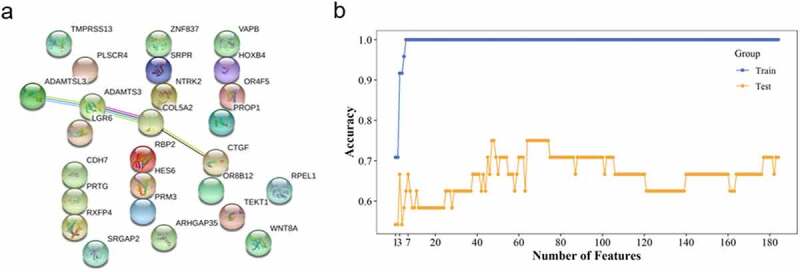
PPI in specific module and SVM-RFE analysis. (a) The PPI of candidate genes; nodes represent genes, edges to interactions. (b) AUC of model conducted with features in ‘test’ and ‘train’ sets was depicted

The PPI of candidate genes was analyzed and depicted by the STRING database (Figure 6a); the result of PPI was integrated with Cytoscape. The PPI indicated one key gene, *ADAMTS3*, played a crucial role with the most interactions during the first period of patients transferring from sepsis to sepsis-induced ARDS. No significantly enriched GO or KEGG terms was found with *P* < 10^−4^.

### 3.7 Potential molecular biomarkers for sepsis and sepsis ARDS

**Figure 7.**
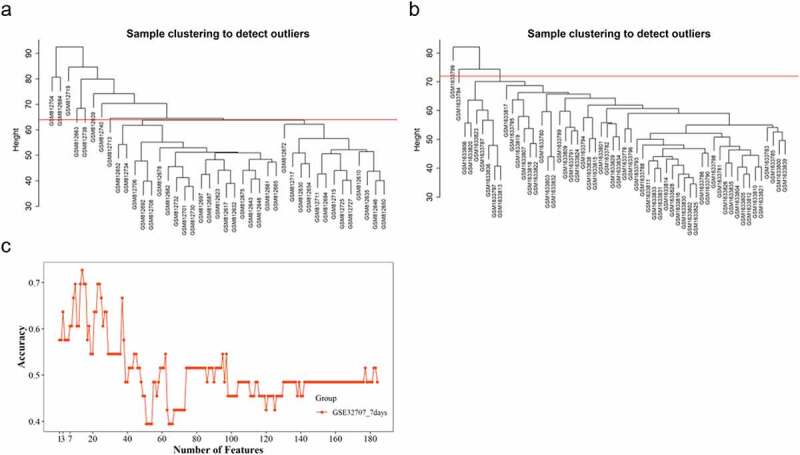
**Validation of selected features**. (a) and (b) represent the cluster tree of samples of samples on 7 days after admission in GSE32707 and GSE66890; samples above the red line will be trimmed. (c) The validation of SVM-RFE with samples (7 days after admission) in GSE32707

To select significant features of sepsis with ARDS, features were detected in ‘train’ set with SVM-RFE method using 194 genes (160 in the opposite model and 34 in the specific model) and validated with the ‘test’ set; the model of 3 genes (*TLCD4, PRSS30P*, and *ZNF493*) and seven genes (*TLCD4, PRSS30P, ZNF493, AGO2, SLC37A3, SLC2A1*, and *RPL11*) showed moderate performance in the ‘test’ group with AUC of 0.67 (Figure 6b). Eight outliers in GSE32707 were identified and cut using a cluster tree; 33 samples, 10 patients with sepsis and ARDS and 23 patients with sepsis alone, were used for validation (Figure 7a). Two outliers – GSM1633784 (ARDS with sepsis) and GSM1633799 (ARDS with sepsis) – were recognized, labeled, and discarded in GSE66890 with the cluster tree; fifty-five samples including 28 sepsis patients and 27 sepsis with ARDS were collected for the validation process (Figure 7b). The model of the top three genes (*TLCD4, PRSS30P*, and *ZNF493*) showed acceptable discrimination in samples on 7 days after admission with AUC of 0.63; however, we didn’t observe the consistent performance of 7 genes (Figure 7c). Three of the top 10 genes, including the first gene, *TLCD4*, were not detected in GSE66890; therefore, we performed validation in GSE66890 using the 2nd to 8th of genes; they failed to identify patients with sepsis and ARDS in GSE66890.

## 4. Discussion

With limited management of sepsis during the past years, it is essential to further elucidate the underlying mechanisms of sepsis and sepsis-induced ARDS to explore the new therapeutic approaches. In this research, we identified the opposite and specific modules related to sepsis and sepsis-induced ARDS via WGCNA. Fifteen genes (*KMO, RPS27A, SPNS2, TUBA1B, BASP1, FBRSL1, AATK, PLK5, LRRC75A, TLCD4, IP6K3, DPY19L2P2, LRRN2, BTLA*, and *ALOX15*) in opposite model significantly differentially expressed in patients with sepsis and ARDS compared to those with sepsis alone. Functional enrichment analysis of genes in the module that were oppositely correlated with sepsis and sepsis-induced ARDS showed that the ribosome-related pathway was significantly enriched (Figure 3). This result is consistent with the previous study in sepsis [20]. *ADAMTS3* was found to be the most active gene in the PPI network in the first periods of patients transferring from sepsis to sepsis induced ARDS (Figure 6a). The adamalysin-thrombospondin (*ADAMTS*) proteinases are a relatively new branch of the metzincin family that contains metalloproteinase, disintegrin, and thrombospondin motifs [21]. It functions in extracellular matrix processing, organogenesis, and hemostasis [22]. *ADAMTS* was considered as a potential biomarker for distinguishing sepsis and sepsis-related ARDS patients, though with poor performance, in the previous research [23]. In our analysis, we didn’t observe significant difference of *ADAMTS* expression in patients with sepsis and ARDS and sepsis alone, indicating the limited potential of *ADAMTS* as biomarker for sepsis-related ARDS and sepsis.

We performed feature selection analysis and found that combination of three genes (*TLCD4, PRSS30P*, and *ZNF493*) and seven genes (*TLCD4, PRSS30P, ZNF493, AGO2, SLC37A3, SLC2A1*, and *RPL11*) showed moderate performance in identifying patients with sepsis and ARDS within 1 day after admission (Figure 6b). We noticed the presence of ribosome-related genes, including *RPL11* and *RPS27*, in the top 20 features. Ribosome, a kind of conserved macromolecular machine, functions as a crucial component in catalyzing protein synthesis; several publications reviewed the significant relation of the ribosome and people’s health and diseases [24,25]. Vary and his coworkers found that the formation of 40S initiation complex was significantly decreased in patients with sepsis compared with healthy people [26]. A recent publication observed a significant enrichment of DEGs in ribosome-related pathway with multiple gene expression profiles [20]. We found the significant enrichment of genes in opposite model in ribosome-related terms and ribosomal genes with critical contribution in identifying patients with sepsis and ARDS form people with sepsis alone, indicating the significance of analyzing the mechanism of ribosome-related pathways in the conversion from sepsis to sepsis-related ARDS. Seven days after admission, the first three genes were consistent in identifying patients with sepsis and ARDS (Figure 7c). However, this potential was not observed when we used another validation data – GSE66890. Though the top three genes showed relatively stable performance in patients within 24 hours and 7 days after admission, the performance was significantly various between two groups (Figure 6b, Figure 7c), which indicates the variety of sepsis and sepsis-related ARDS; more expression experiments need to be done for potential biomarker exploration.

## 5. Conclusion

In conclusion, we constructed networks between sepsis patients and sepsis-related ARDS patients with WGCNA. Function analysis of candidate genes and feature selection analysis revealed the crucial role of ribosome-related genes in the development of sepsis and sepsis-related ARDS. Three genes–*TLCD4, PRSS30P*, and *ZNF493* indicated moderate performance in distinguishing sepsis-induced ARDS from sepsis.

## Supplementary Material

Supplemental Material

## Data Availability

The datasets used and/or analyzed during the current study are available from the corresponding author on reasonable request.
